# Characterization of Lignocellulolytic Activities from a Moderate Halophile Strain of *Aspergillus caesiellus* Isolated from a Sugarcane Bagasse Fermentation

**DOI:** 10.1371/journal.pone.0105893

**Published:** 2014-08-27

**Authors:** Ramón Alberto Batista-García, Edgar Balcázar-López, Estefan Miranda-Miranda, Ayixón Sánchez-Reyes, Laura Cuervo-Soto, Denise Aceves-Zamudio, Karina Atriztán-Hernández, Catalina Morales-Herrera, Rocío Rodríguez-Hernández, Jorge Folch-Mallol

**Affiliations:** 1 Facultad de Ciencias, Universidad Autónoma del Estado de Morelos, Cuernavaca, Morelos, Mexico; 2 Centro de Investigación en Biotecnología, Universidad Autónoma del Estado de Morelos, Cuernavaca, Morelos, México; 3 Centro Nacional de Investigación Disciplinaria en Parasitología Veterinaria, Instituto Nacional de Investigaciones Forestales Agrícolas y Pecuarias, Cuernavaca, Morelos, Mexico; 4 Facultad de Ciencias Biológicas, Universidad Autónoma del Estado de Morelos, Cuernavaca, Morelos, Mexico; Missouri University of Science and Technology, United States of America

## Abstract

A moderate halophile and thermotolerant fungal strain was isolated from a sugarcane bagasse fermentation in the presence of 2 M NaCl that was set in the laboratory. This strain was identified by polyphasic criteria as *Aspergillus caesiellus*. The fungus showed an optimal growth rate in media containing 1 M NaCl at 28°C and could grow in media added with up to 2 M NaCl. This strain was able to grow at 37 and 42°C, with or without NaCl. *A. caesiellus* H1 produced cellulases, xylanases, manganese peroxidase (MnP) and esterases. No laccase activity was detected in the conditions we tested. The cellulase activity was thermostable, halostable, and no differential expression of cellulases was observed in media with different salt concentrations. However, differential band patterns for cellulase and xylanase activities were detected in zymograms when the fungus was grown in different lignocellulosic substrates such as wheat straw, maize stover, agave fibres, sugarcane bagasse and sawdust. Optimal temperature and pH were similar to other cellulases previously described. These results support the potential of this fungus to degrade lignocellulosic materials and its possible use in biotechnological applications.

## Introduction

For many years, extremophile microorganisms were described exclusively in the Archae and Eubacteria domains [1]. These peculiar organisms have been found and studied in environments with very extreme conditions such as temperature, pressure, pH, salinity and radiation, among others. Physiological and metabolic studies on extremophile microorganisms derived in the development of biotechnological applications with incidence in the agricultural, pharmacological and environmental industries [2]–[6]. The description of microbial diversity in extremophile ecosystems has been of great interest for a long time until our days. However, the report of eukaryotic extremophiles took many years.

In particular, environments with high concentrations of salt, called saline and hypersaline environments, have been attractive ecosystems for studying the structures of microbial communities that inhabit them [7]–[9], since sodium chloride has been considered an effective microbicide. For example, treating food with salt is an effective method of preserving meat since ancient times [10]. The isolation and characterization of microorganisms in habitats with over 1 M NaCl is important for the identification of metabolites and/or robust proteins with potential industrial applications, and to understand the cellular physiology, molecular biology and biochemistry that support the survival of these organisms under extreme conditions [11]–[13].

Numerous studies have described halophilic bacterial genera in hypersaline ecosystems [5], [8], [14]–[21]. However the first report of a fungus isolated from a solar saltern appeared in the year 2000 [22]. Since then, many studies on biodiversity and physiology have reported the characterization of halophilic fungi present in saline and hypersaline ecosystems. Many species of Ascomycetes, and some Basidiomycetes, have been described with the ability to grow in these environments [22], [23]. Currently, the presence of fungi in the structure of microbial communities that compose some ecosystems with extreme salinity conditions has been described [24]. Until 2009, only 10 orders of fungi known to tolerate low water activity (a_w_) due to high salt concentrations have been reported [22].

Among the most studied halophilic fungal genera are *Cladosporium* [22], [23], [25], *Wallemia*, *Scopulariopsis*, *Alternaria* [24], [26] and some species of *Aspergillus* and *Penicillium* [23]. Particularly in the *Aspergillus* genera, *A. niger, A. sydowwi, A. flavus, A. tubingensis* and *A. versicolor* have been isolated and described as halotorelant and halophile fungi as part of the hypersaline environments [22]. The study of these fungi has allowed the characterization of halophilic enzymes with interesting properties [27]. For example, cellulases and xylanases have been described from halophilic microorganisms, filamentous fungi included, with interesting biochemical properties such as activity in the presence of high salt concentrations, at acidic pH, or in ionic liquids [11], [13], [28]–[33]. In general, halophilic microorganisms can be isolated from the sea, saline or hypersaline lakes, solar salterns, and salted foods, among other habitats with high concentrations of salt.

It was assumed for a long time that extremophile microorganisms were strictly growing in extreme conditions; however this assumption was proved to be false because there are many examples of extremophiles isolated from non-extreme environments [34]–[36]. In this way, some reports demonstrate that halophilic and/or halotolerant microorganisms are not restricted to saline or hypersaline habitats and can be found almost everywhere in non-saline environments [37]–[46]. The general principles of microbial ecology, the physiological plasticity and metabolic versatility of the microorganisms do not impede the isolation of halophile and/or halotolerant organisms from non-saline environments.

The isolation of halophile and/or halotolerant fungi able to grow in lignocellulosic materials is a very interesting source for the search of industrially useful enzymes, potentially capable of producing fermentable sugars from agricultural wastes. One of the major limitations of using enzymes in industry is obtaining robust biocatalysts capable of operating under rigorous industrial conditions. Because of this demand, the study of halophilic and halotolerant microorganisms is an appropriate strategy for the characterization of enzymes such as cellulases, esterases, lipases and xylanases with potential application in biorefineries. Sugarcane bagasse is considered a recalcitrant, high cellulose content agroindustrial waste. The microbial communities growing on sugarcane bagasse, especially filamentous fungi must have efficient enzymes to obtain energy and carbon from lignocellulose degradation. The aims of this work were to characterize a filamentous fungus isolated from sugarcane bagasse with the potential to grow in concentrations of sodium chloride higher than 1 M, and to analyse the lignocellulolytic activities it produces.

## Materials and Methods

### Isolation and preservation of microorganisms

Three grams of non-sterile sugarcane bagasse (as the main carbon source and from where the microorganisms were isolated) were mixed with 2.5 g of sterile soil (as a source of organic matter) into 1000 mL Erlenmeyer flasks containing 250 mL of Vogeĺs medium as described in [47] and a final concentration of 2 M NaCl. Fermentation was performed at 25°C and 150 rpm for 30 days. Primary isolation of cellulolytic microorganisms was done taking one mL of the sugar bagasse culture and serial dilutions were performed up to 10^−10^. Two hundred µL of each dilution were inoculated in Petri plates containing Vogeĺs medium supplemented with 2% carboxymethylcellulose (CMC, Sigma Catalogue No. C5678) and 0.5 M NaCl. The cultures were incubated for 10 days at 30°C and were observed daily for secondary isolation of cellulolytic bacteria and/or fungi. A fungus was isolated in Potato Dextrose Agar (PDA) and Saboraud agar media (both from DIFCO). This fungus was stored at 4°C in saline solution (0.5% NaCl) supplemented with glycerol (20%). Isolates were performed in triplicate to achieve representativeness of the obtained microbial populations.

### Identification of a fungal moderate halophilic strain

Mycelium from a fungus growing on PDA plates was collected after 10-days for genomic DNA isolation according to a previously reported method [48]. For identification, we analysed molecular markers previously described to be distinctive to filamentous fungi. A fragment of the 18S ribosomal DNA was amplified by PCR using primers nu-SSU-0817 and nu-SSU-1536 as described by Bornenman and Hartin [49]. Also, regions of the 28S large sub-unit RNA gene (D1–D2) and internal transcribed spacers 1 (ITS1 region) were amplified. These regions have been particularly useful for the molecular identification of fungi. The primers and conditions used for these PCR reactions have been previously described by Peterson [50] and Hinrikson *et al.* [51], respectively.

Amplicons were analysed in 1% agarose gel electrophoresis in 1x TBE buffer [52] and purified from the agarose gels using a commercial gel extraction kit (Fermentas Catalogue No. K0513) and sequenced in both directions using the same primers used for the amplification. Sanger sequencing was performed at the Sequencing Unit of the Instituto de Biotecnología of the Universidad Nacional Autónoma de México.

The sequences were analysed using the website of the National Centre for Biotechnology Information (NCBI) (www.ncbi.nih.gov). BLAST search was performed to determine similar sequences. Phylogeny studies allowed defining the relationship of these sequences with those obtained in the BLAST analysis. Phylogenetic analysis was performed online with the server Phylogeny.fr (www.phylogeny.fr). This platform considers various bioinformatics programs to reconstruct a robust phylogenetic tree from a set of sequences. These tools allowed identification of the fungal strain considering molecular criteria.

Micromorphological identification was performed to complete a polyphasic vision for the microbial identification. Size, shape and grouping of conidiophores, morphological aspects of the colony, and the fungal hyphae were analysed. The aspect of the colony on different media (PDA, Saboraud agar and Malt extract agar (MEA, from DIFCO) was also considered.

### Growth Rate Determination

Specific growth rate of the fungal colony (expressed as mm/day) was determined as follows. Plugs of 7 mm of diameter obtained from a fungal pre-culture in Vogel’s medium supplemented with 2% CMC were inoculated in the same medium at different temperatures (28, 37, and 42°C) or supplemented with NaCl (0, 0.5, 1, 1.5, 2 or 3 M, final concentration). The diameter of the colony was measured every 24 hours for 10 days. Experiments were performed in triplicate for subsequent statistical analysis of data.

### Solid-state Fermentation

Solid-state fermentations were performed using the following autoclaved substrates: agave fibre (*Agave fourcroydes*), sugarcane bagasse (*Saccharum officinarum*), maize stover (*Zea mays*), wheat straw (*Triticum aestivum*), and pine sawdust (*Pinus sylvestris*). Erlenmeyer flasks of 500 mL including 2 g of each substrate were inoculated with two plugs of 7 mm of diameter of the fungal strain previously grown on PDA plates. Humidity in the system was maintained by adding 20% (w/v) of distilled water to the solids. Fermentation was allowed to take place at 28°C for 10 days. Subsequently, soluble fermentation products were collected in 10 mL in 60 mM citrate buffer pH 5.

### Enzymatic activity determinations

Cellulase activity was determined qualitatively and quantitatively. For qualitative determinations, seven-day cultures of the fungus grown on agar Vogel’s medium supplemented with 2% CMC and NaCl (0, 0.5, 1, 1.5, 2 M, final concentration) at 28°C were set up. Petri dishes with the colonies were then inundated with approximately 15 mL of Congo red (1% diluted in distilled water) for 10–15 minutes. Subsequently, these dishes were washed three times with approximately 15 mL of a 1 M NaCl solution. Discoloration halos around the colony indicated the degradation of cellulose due to the production of cellulases [53], [54]. Determinations were performed in triplicate.

For quantitative determination of cellulase activity, plugs of 7 mm of diameter from pre-cultures of the fungus grown in Vogeĺs medium with 2% CMC were inoculated in 500 mL Erlenmeyer flasks with 100 mL of the same medium plus NaCl (0, 0.5, 1, 1.5, 2 M). The flasks were incubated for 9 days at 28°C and 150 rpm. Enzymatic activity and protein concentration were determined every 24 h from 2 mL of the supernatants of these liquid cultures. Enzymatic activity was assessed by the production of reducing sugars from polymeric substrates using the 3,5-dinitrosalysilic acid (DNS) assay described by Miller [55]. Protein concentration was determined by the Lowry method [56]. Enzymatic specific activity is expressed as IU/mg protein.

For cellulases activity measurements, CMC (2%) dissolved in 50 mM citrate buffer pH 5 was used as a substrate. The enzymatic reaction contained 200 µL of supernatant, 300 µL of 50 mM citrate buffer pH 5 and 500 µL of substrate solution. The reaction mixtures were incubated at 50°C for 30 minutes. Briefly, 50 µL aliquots were taken every 5 minutes (after adding the supernatant to the reaction mixture) up to 45 minutes and then mixed with 50 µL of a DNS solution, boiled for 5 minutes and immediately cooled on ice for 5 minutes. Finally 500 µL of water were added and absorbance measured at λ 540 nm in a spectrophotometer (BioMate, ThermoSpectronic). Reducing sugars concentration was extrapolated from a glucose standard curve ranging from 0.1 to 2 mg/mL; concentration values were plotted against time, and the slope was calculated to determine the velocity of the reaction. Concentration of released reducing sugars *vs.* time was used to calculate enzymatic activities, where 1 IU is defined as 1 µmol of glucose equivalent released per minute, under the assayed conditions. For specific activity calculation, protein concentration in mg/mL was determined against a bovine serum albumin (BSA) standard curve [57]. Xylanases activity was measured in a similar way using 2% oat xylan (Sigma Catalogue No. X0627) as substrate. The absorbance readings were compared against a standard curve of xylose (0.1 to 2 mg/mL).

MnP activity was determined spectrophotometrically (λ 270 nm) by following the formation of Mn^3+^−malonate complex at pH 4.5 in 50 mM sodium malonate buffer with 0.5 mM MnSO_4_ [58]. To start the reaction, H_2_O_2_ was added to a final concentration of 0.1 mM [59]. The reaction was followed for 30 seconds at room temperature. ΔAbs min^−1^ was converted to UL^−1^ using the malonate extinction coefficient of 11 590 M^−1^cm^−1^ [58]. MnP specific activity is expressed as IU/mg protein.

Laccase activity was monitored by oxidation of 1 mM of 2,2′-azino-bis(3-ethylbenzthiazoline-6-sulfonic acid) (ABTS) in acetate buffer (pH 4.5), measuring formation of the cation radical (ε436 = 2.9×104 M^–1 ^cm^–1^). The reaction mixture volume was 1 mL and was incubated for 30 minutes at room temperature. One unit of laccase activity is defined as the amount of enzyme catalysing the oxidation of 1 µmol of substrate per minute. ΔAbs min^−1^ was determined at λ 420 nm using spectrophotometer (BioMate, ThermoSpectronic).

Lipase/esterase and esterase activities were assessed using 2-naphthyl acetate (Sigma Catalogue No. N6875) and 4-nitrophenyl acetate (Sigma Catalogue No. N8130) as substrates, respectively. A stock solution (250 µM) of 2-naphthyl acetate was prepared in phosphate buffer saline (PBS) pH 6.5, while a stock solution of 4-nitrophenyl acetate (300 µM) was prepared in potassium phosphate buffer pH 6. The reaction volume was 1 mL and it was followed for 30 minutes at room temperature. The determination of these activities was measured spectrophotometrically at λ 538 nm for detection of 2-naphtol and at λ 410 nm for 4-nitrophenol products of enzymatic hydrolysis of lipase/esterase and esterase respectively, using the molar extinction coefficient for each substrate (23 598 M^−1^cm^−1^ for 2-naphthol and 17 700 M^−1^cm^−1^ for 4-nitrophenol). Lipase/esterase and esterase specific activities are expressed as IU/mg protein.

To determine all activities we plotted the absorbance values *vs* time, and then calculate the value of the slope of the best fitted line to the experimental points. Previously, we determined the protein concentration as described above. Finally we calculated the volumetric enzymatic and specific enzymatic activities (express as IU/mg protein).

Triplicate independent assays were performed and three readings for each sample were taken in all cases. A BioMate, ThermoSpectronic spectrophotometer was used for all the measurements.

### Zymograms

Zymograms were performed to identify cellulase and xylanase activities from the supernatants of the fungal culture on Vogel’s medium supplemented with 2% CMC and NaCl (0, 0.5, 1, 1.5, 2 M), or from soluble products recovered from the solid-state fermentations. Zymograms were performed as described in Quiroz-Castañeda *et al.* [57]. Briefly, 50 µg of protein were loaded per lane in the gels for the experiments in media containing NaCl, or 30 µg from the samples of the solid-state fermentation experiments. For cellulases, the gels were embedded with 2% CMC, while 2% oat xylan was used in the gels for xylanases. *Trichoderma viride* cellulase (Sigma Catalogue No. 1794), and xylanase (Sigma Catalogue No. 3876) were included as controls. Once the native-PAGE electrophoresis was carried out, gels were incubated with a 1% Congo red solution (in water) for 30 minutes at room temperature and then washed 3 times with a 1 M NaCl solution. The cellulase activity developed as clear bands. The molecular weight of the bands was estimated against a protein marker (Fermentas Catalogue No. 26612).

### Optimal temperature and pH of cellulase activity

Enzyme reactions were performed as described earlier at different incubation temperatures (20, 30, 40, 50, 60, 70 and 80°C) in 50 mM sodium citrate buffer, pH 5. Different pH conditions ranging from 3 to 8 were tested at 50°C in citrate or phosphate buffer depending of the pH tested. All measurements were determined in triplicate.

### Thermal-stability of the cellulase activity

Cellulases thermostability was determined by incubating 200 µL of supernatant for 60 minutes at the following temperatures: 30, 40, 50, 60 and 70°C. Subsequently, the samples were cooled on ice for 5 minutes and the residual activity was determined as described before (at 50°C and pH****5). All measurements were performed in triplicate.

### Statistical calculations

For statistical treatment of experimental data, the arithmetic mean and the standard deviation were calculated. Simple classification ANOVA tests were applied to determine significant differences between the different cases. Firstly, the assumptions of ANOVA were revised: analysis of homogeneity of variance (Hartley-Cochran-Bartlett test) and normal distribution (Kolmogorov-Smirnov and Lilliefors tests) were performed [60]. Subsequently ANOVAs were conducted to demonstrate the similarities or differences between the data of the population of samples. Finally, a post hoc analysis that defines the order of the differences found in the ANOVAs was developed. The Fisher LSD, Tukey HSD and Duncan tests were considered for the post hoc analyses [60], [61]. The use of these three tests ensures greater statistical robustness of the proposed analysis. All statistical calculations were performed in STATISTICA (last version for computers).

## Results and Discussion

### Isolation of microorganisms

**Figure 1:**
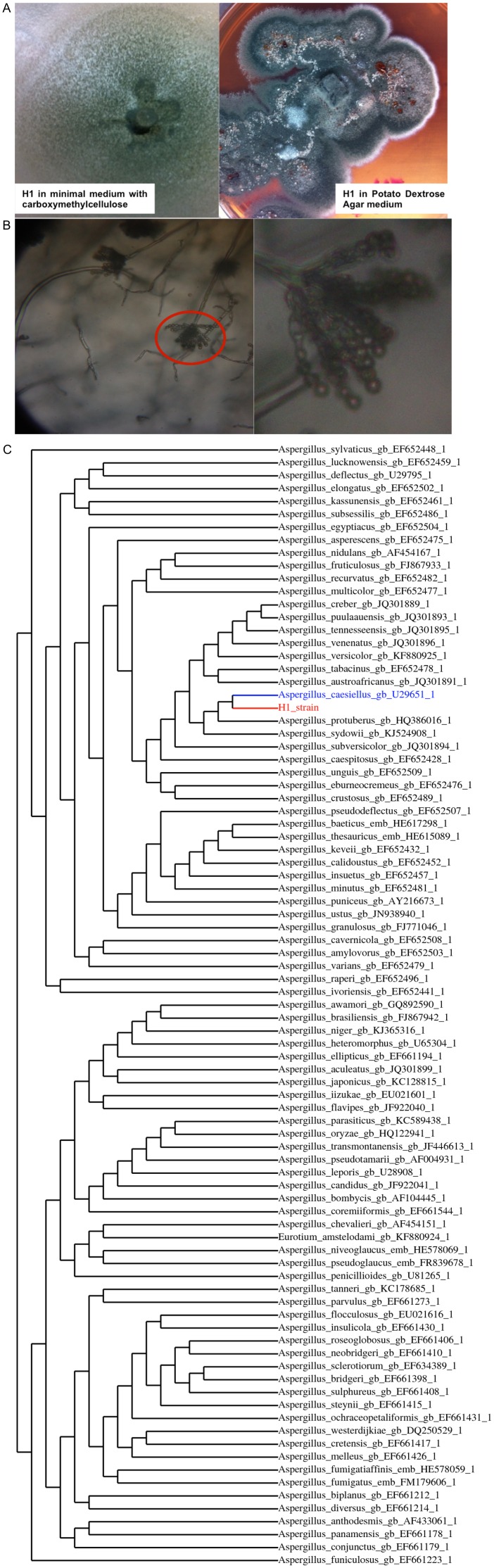
(A) *A. caesiellus* grown in Vogel’s medium supplemented with CMC (2%) with 0.5 M NaCl and PDA medium. (B) Microcultures of *A. caesiellus* in Saboraud Agar medium. (C) Molecular phylogeny for the D1–D2 domain of 28S rDNA gene.

No bacterial or yeast growth was detected in the primary isolation of microorganisms, whereas a well-represented filamentous fungus was isolated in all serial dilutions up to 10^−5^. This fungal strain was named H1 and showed the capacity to grow in 0.5 M NaCl and CMC as a carbon resource. During the secondary isolation in selective culture media (Sabouraud Agar or PDA added with 0.5 M NaCl) the fungus grew as a dark green colony on the aerial mycelium and brownish-grey in the vegetative mycelium (Figure 1). The production of metabolites secreted as dark brown and bright yellow pigments was evident. The selection forces we used to isolate microorganisms able to degrade lignocellulosic material under high salinity conditions were a complex source of carbon and energy (sugarcane bagasse) and 2 M NaCl. High NaCl concentrations inhibit the growth of most microorganisms; this ratio may explain the very low representation of the microbial population obtained during primary isolation taking in account that it came from a non-saline environment [1], [62]–[64].

### Identification of H1

Macroscopic observations of the colony and micromorphological characteristics (hyphae, conidiophores and conidial shape) suggested that the isolate belonged to the genus *Aspergillus* (Figure 1).

Light green grainy colonies were observed in Vogel’s medium with 2% CMC and 0.5 M NaCl, while medium dark green colonies were observed in PDA (Figure 1A). Microscopic examination of the culture mycelia showed columnar conidiophores of approximately 350 µm in length with vesicles 20–35 µm in diameter and pale conidia with globose and subglobose and smooth spores. Spinose or roughened conidia were never observed. The conidia length was between 5 and 6 µm while the conidia width was approximately 3 µm (Figure 1B). These observations coincide with the characteristics described for *Aspergillus caesiellus* in the CBS-KNAW Fungal Biodiversity Centre (www.cbs.knaw.nl).

To confirm the identity of this strain, molecular markers for H1 strain were analysed. We chose to amplify a fragment of the 18S rDNA, the D1–D2 domain (28S rDNA) and the ITS1 region; the determined sequences were annotated in the GenBank with the accession numbers KJ476140, KJ476141 and KJ476142, respectively. BLAST DNA sequences analyses showed similarity with various species of *Aspergilli* demonstrating that H1 belongs to the *Aspergilli* genus.

Many molecular markers with taxonomic value have been described in the genus *Aspergillus*. These include the mitochondrial cytochrome *b* gene [65]–[67], a putative aflatoxin pathway regulatory gen (*aflR*) [68], the DNA topoisomerase II gene (*TOP2*) [69], the β-tubulin gene [70] and different rDNA gene regions [71]. Among the regions of the rDNA genes the most representative are the 5′end of the large-subunit 28S rDNA gene (D1–D2 region) [50] and the internal transcribed spacers 1 and 2 (ITS1 and ITS2) regions between the small- and large-subunit rDNA genes [51], [71], [72]. It has been reported that for the *Aspergilli*, the ITS1 and D1–D2 regions are the most variable and therefore the most useful for molecular identification of this genus species [51].

To define the species related to H1 strain, phylogenetic reconstructions based on the amplified sequences for each of the molecular markers were studied. Sequences with more than 80% identity and 70% coverage obtained through Blastn analysis were considered to reconstruct the phylogenies. The phylogenetic analysis of the 18S rDNA fragment was inconclusive for species identification. In this case, the H1 sequence does not group with any particular species of *Aspergillus,* although the closest relative to H1 was *Aspergillus versicolor* (Figure S1). On the other hand, the analysis of molecular phylogenies for sequences of the ITS regions were also not conclusive because H1 strain was grouped individually on a single branch of the phylogenetic tree (Figure S1). In this case, the most closely related species was *Aspergillus niger* (Figure S1).

Phylogeny analysis obtained for the sequences of the D1–D2 domain allowed us to propose the identity of strain H1 as *Aspergillus caesiellus*. Numerous studies show this region as very useful for identification filamentous fungi, not only for *Aspergillus* species [73]–[79]. In this case, our sequence was grouped only with a reference strain of *A. caesiellus* (*A. caesiellus* NRRL a-14879 annotated in the CBS-KNAW Fungal Biodiversity Centre). In this phylogeny it was found that *Aspergillus sydowii* and *Aspergillus protuberus* were the closest species to H1 (Figure 1C).

*A. sydowii* and *A. protuberus* are classified in the *Aspergillus* section *Versicolores* which was established as the *A. versicolor* group by Thom and Churche [80] and it was after reconsidered by Thom and Raper [81]. *A. protuberus* does not produce soluble pigments or exudates on MEA and its colonies show reverse light pinkish yellow to pinkish yellow colour. Moreover it presents conidia with finely roughened walls that can also be ellipsoidal to pyriform [82]. *A. sydowii* either produce soluble pigments or exudates on MEA and exhibit colonies with unpigmented reverse to brownish pink colour [82]. *A. sydowii* isolates growing specifically on Saboraud media appear as dark green colonies with a white fringe (www.thunderhouse4-yuri.blogspot.ie) and excrete purple pigments [83]. Its colonies on PDA are blue-green colour, reverse reddish and often with reddish exudates. Red-brown colour in PDA and Czapeks agar media is a very distinctive characteristic of *A. sydowii* [83], [84]. Besides, the micromorphological characterization of this species confirms spherical, and very echinulate or spinose (rough, jagged texture) conidia [83], [84].

Strain H1 shows significant differences with all the above descriptions because the presence of abundant exudates and dark soluble pigments (non-reddish and non-purple) on Saboraud agar, MEA and PDA media was detected and microculture conidia with the previously described characteristics for *A. sydowii* and *A. protuberus* were not observed. Additionally the aspect of the H1′s colony does not coincide with those described for the previous two species. Cultural and morphological criteria support the molecular identification proposal for strain H1 because they coincide phylogenetically with H1 and the rooted closest species. Systematics and taxonomy of fungi, especially for the genus *Aspergillus* spp., continue up to day using morpho-culture characters for the distinction between related species [82], [85]–[88]. On the other hand, *A. protuberus* cannot grow at 37°C [82], while H1 showed thermotolerant behaviour (see next section). In our case, it was critical to evaluate the colony texture, colour on the nutrient media, excretions of exudates and soluble pigments and the characteristics of the conidia to conclude the taxonomic identification. Thus, attending molecular and micromorphological criteria H1 strain was identified as *Aspergillus caesiellus*. We consider that the identification of the strain H1 is robust and conclusive according to the current criteria for identification of filamentous fungi. The polyphasic approach for the identification of microorganisms ensures better taxonomic conclusion according to several studies [89], [90].

*A. caesiellus* has been poorly studied. There are very few reports about the biology and physiology of this filamentous fungus. Some of these studies have reported the potential of this fungus to produce keratinase [91], amylases [92], and invertases [93]. However, little is known about the biology of this species; besides the original report, only few studies describe the isolation of *A. caesiellus* from different sources including air and dust, [94], a marine sponge [95], and chicken litter [91]. *A. caesiellus* has also been reported as a pathogen fungus or opportunistic pathogen [96], [97].

To the best of our knowledge, this is the first report describing the isolation of *A. caesiellus* from samples of lignocellulosic material. This strain is able to grow in different NaCl concentrations and tolerant up to 2 M NaCl. Phylogenetically related species such as *A. versicolor* and *A. sydowii* have been described as halotolerant and halophilic by some authors [22], [98]–[101]. These species were related to H1 strain in the constructed molecular phylogenies, a finding that also supports its molecular identification. This study demonstrates the flexibility and plasticity of microbial physiology and the possibility to isolate halotolerant and/or halophilic microorganisms from non-hypersaline environments. The assumptions of microbial ecology are general and valid but not absolute in these terms.

### *A. caesiellus* H1 is a moderate halophile and thermotolerant fungus

**Figure 2:**
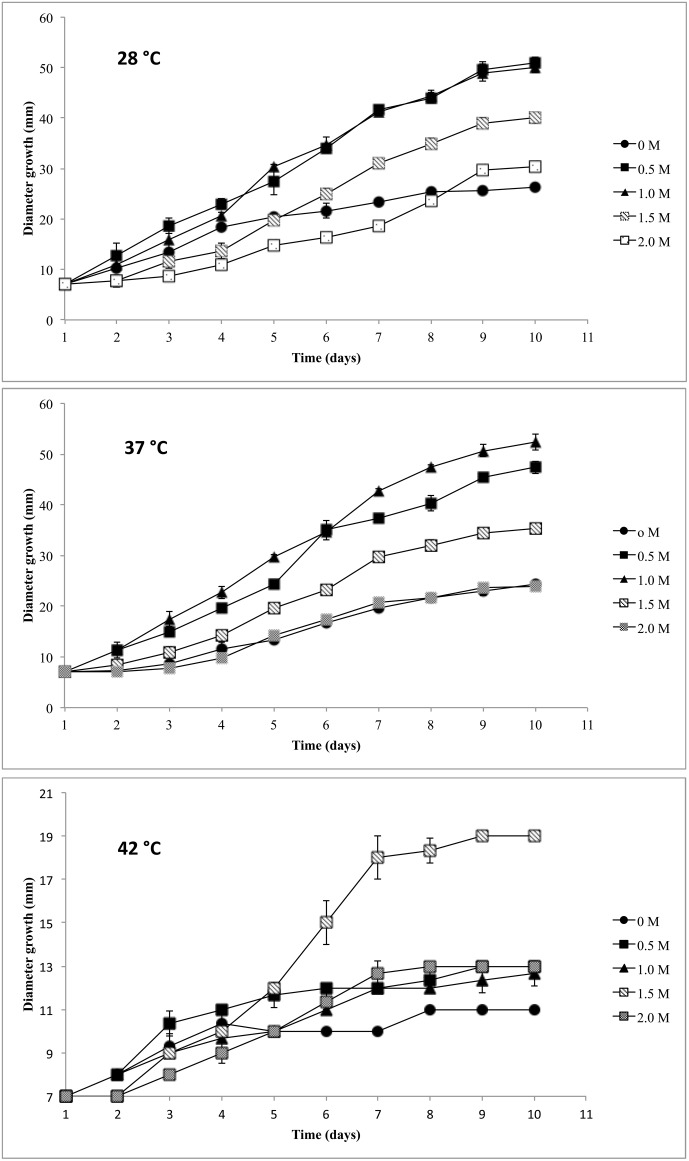
Growth curves of H1 at different temperatures and NaCl concentrations.

The growth curves of *A. caesiellus* H1 showed statistically significant greater growth rates in the presence of NaCl as compared to those without NaCl for the three temperatures tested (28, 37 and 42°C) (Figure 2). H1 growth was inhibited only in 3 M NaCl. The higher specific growth rate was obtained in cultures of H1 at 37°C and 1 M NaCl. Similar specific growth rates were observed under conditions of 0.5 and 1 M NaCl in experiments at 28°C, while at 42°C, the higher specific growth rate is achieved in the culture conditions of 1.5 M NaCl (Table 1). These results show that the H1 is also a thermotolerant strain. Thermotolerant organisms can grow in a range of temperatures above 40°C [102], [103].

**Table 1:** Specific growth rate (mm/day) of the strain H1 at different temperatures and NaCl concentrations.

NaCl (M)	Growth rate 28°C	Growth rate 37°C	Growth rate 42°C
0	2.17±0.03^d^	2.17±0.07^e^	0.39±0.03^d^
0.5	5.12±0.22^a^	4.80±0.02^b^	0.62±0.03^c^
1.0	5.22±0.02^a^	5.40±0.10^a^	0.63±0.07^c^
1.5	4.18±0.14^b^	3.59±0.07^c^	1.62±0.04^a^
2.0	2.81±0.05^c^	2.28±0.10^d e^	0.80±0.01^b^

Different letters indicate different statistical orders.

There is still much controversy about the definition of extremophile microorganisms. However, one of the most accepted, defines extremophile organisms as those showing optimal growth parameters in different environmental conditions than those normal for humans [104]. In the particular case of halophile microorganisms, there are also disputes about which is the best definition. Certainly it is even difficult to define the boundaries between the concepts: halotolerant and halophile. The establishment of the difference is harder in fungi, even up to date the limits between halotolerant and halophilic strains is not outspoken [46]. However, these definitions are more than fifty years old. Larsen [105] and Kushner and Kamekura [106] introduced three important definitions: moderate halophiles, extreme halophiles and halotolerant.

Halotolerance and halophilia are very clearly defined for Bacteria and Archae. Prokaryotes are classified as obligated halophiles when they require NaCl for optimal growth. Bacteria can be grouped in two categories according with the NaCl concentration required: extremely halophilic or moderately halophilic. In general, definitions mark off extremely halophilic bacteria when they require NaCl concentrations of 2.5–5.2 M (15–30%) for their optimal growth rate, while moderate halophiles grow optimally in media containing 0.5–2.5 M (3–15%) NaCl [107]–[110]. Some authors have defined for convenience halophilic bacteria as microorganisms that form colonies on agar plates with 20% added NaCl [44]. General definitions as that of Madigan *et al.* [104] classify halophile organisms as those requiring high concentrations of NaCl (1.5 M) to growth optimally. In contrast, halotolerant microorganisms are defined as those showing optimal growth parameters in the absence of salt, but can survive in not-common NaCl concentrations [111], [112].

For fungi, the term halophile was introduced for first time in 1975 to describe xerophilic species inhabiting food that showed superior growth in media with NaCl as the controlling solute [113]. Gunde-Cimerman *et al.* [22], [24] proposed as halophilic or xerophilic fungi those growing well at a_w_ of ≤0.85, corresponding to 17% NaCl (3 M) or 50% glucose added to their growth medium. In the same review [22] the authors noted that these fungi have different halophilic characteristics when they are compared with the majority of halophilic prokaryotes. Plemenitaš *et al.* [114] described that most halophilic fungi do not require salt for viability because they can show adaptive properties to grow in any salinity range, from freshwater to saturated NaCl concentrations.

Thus, there are several criteria to classify microorganisms attending their growth and survival in different NaCl concentrations. We consider that *A. caesiellus* H1 can be classified as a moderate halophile fungus because, although it does not exhibit the best growth rate at NaCl concentration higher than 17%, it has greater specific growth rates in the presence of high salt concentrations than those in the absence of NaCl. However, the H1 strain was not isolated from a hypersaline environment and is capable of growing without NaCl in the medium. Our proposed classification attends the halotolerance and halophilic concepts reviewed by different authors. This shows that moderate extremophile microorganisms can live in environments that are not extreme (see Introduction).

This study is the first report of a moderate halophile strain of *A. caesiellus*. Other species have been described in the genus as halophile/halotolerant, for example, *A. versicolor, A. sydowii, A. flavus*, etc. Some species, such as *A. versicolor and A. sydowii* have been found as part of the fungal microbiota of hypersaline environments [22], [99], [100], [115]–[117].

The physiology and metabolism of microorganisms with the ability to grow in high concentrations of NaCl ensure a number of applications of biotechnological interest [118]–[121]. In particular, the study of the ability of *A. caesiellus* H1 for degradation of lignocellulosic material became an attraction for our research group. H1 is a strain isolated from sugarcane bagasse, able to grow at a range of NaCl between 0.5 and 2 M and uses cellulose as the sole source of carbon and energy. These qualities point to *A. caesiellus* H1 as a good candidate to study some of its lignocellulolytic enzymes.

### Cellulases activity

**Figure 3:**
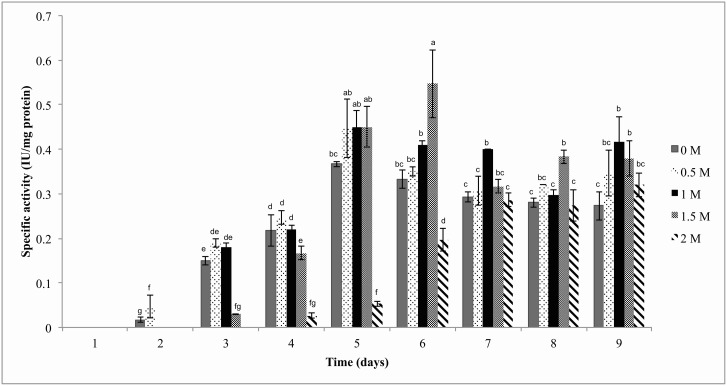
Cellulase activity of H1 cultures on CMC (2%) as the sole carbon source at different NaCl concentrations.

Cellulase enzymatic activity was observed at its highest level within the sixth day of culture in 1.5 M NaCl, using CMC as only carbon source at 28°C (Figure 3). From the fifth day on, cultures of H1 in the presence of varying salt concentrations showed higher cellulase specific activity compared to cultures in the absence of NaCl with exception of the 2 M condition, which from day 7 on anyway showed similar activities to the control culture (without NaCl) (Figure 3). To the best of our knowledge this is the first report of cellulase activity for *A. caesiellus*.

**Figure 4:**
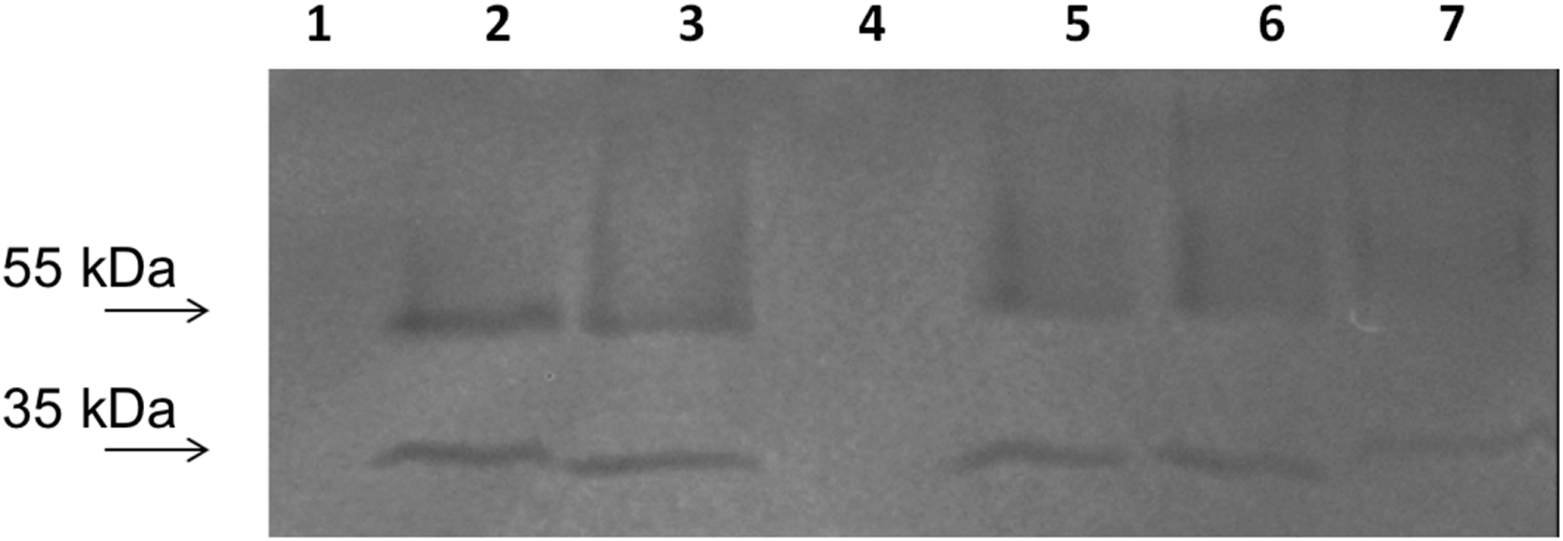
Zymogram for detection of extracellular cellulases from liquid cultures of strain H1 at different NaCl concentrations in CMC (2%) as the sole carbon source. Lane 1: Control culture in 2% glucose without NaCl. Lane 2: Culture without NaCl. Lane 3: Culture with 0.5 M NaCl. Lane 4: No sample charge. Lane 5: Culture with 1 M NaCl. Lane 6: Culture with 1.5 M NaCl. Lane 7: Culture with 2 M NaCl. Arrows indicate the position were the molecular weight markers migrated.

Zymograms of supernatants of *A. caesiellus* H1 cultures in CMC as a carbon source showed two isoforms for cellulases (of around 50 and 35 kDa), regardless of the NaCl concentration (Figure 4). The 50 kDa activity band diminishes at high salt concentrations (1.5 and 2 M), since the amount of protein loaded was the same for all the lanes. We were not able to detect any bands in glucose grown cultures in this experiment suggesting that cellulase expression is repressed by glucose (Figure 4, lane 1). These results indicate that cellulases from *A. caesiellus* H1 are capable of functioning at concentrations up to 2 M NaCl, since growth is supported in this condition with CMC as a sole carbon source.

### Solid-state fermentation of natural substrates by H1

**Figure 5:**
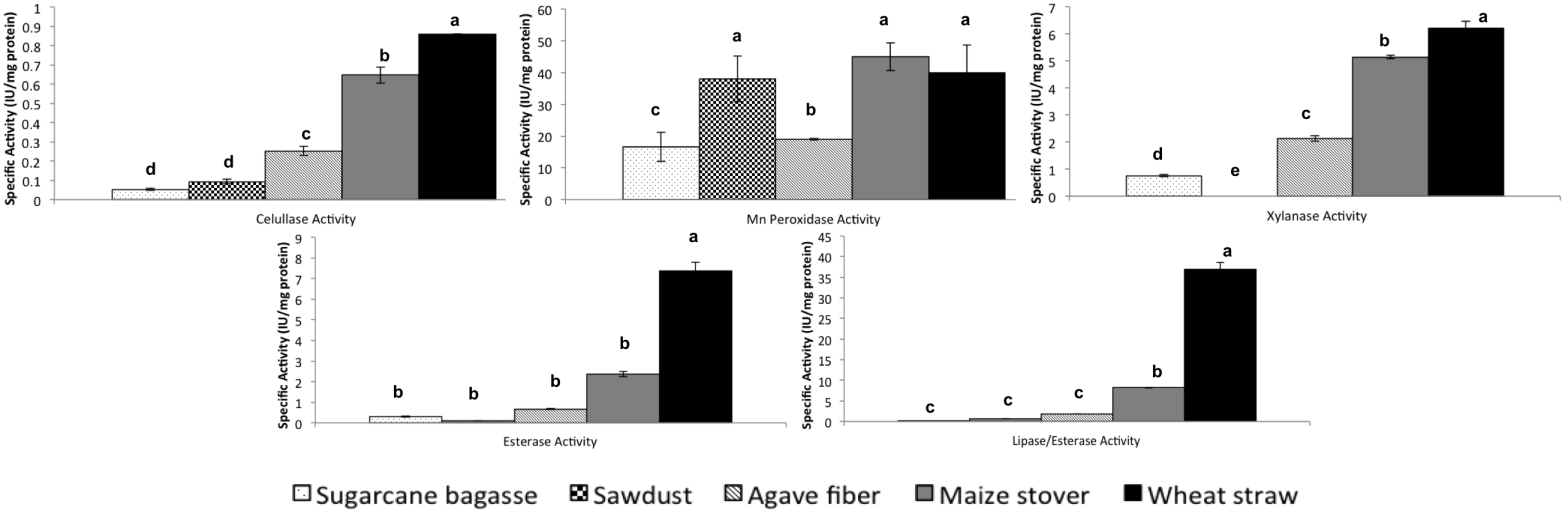
Enzymatic activities from solid-state fermentation of H1 in different substrates. Different letters indicate different statistical orders.

**Figure 6:**
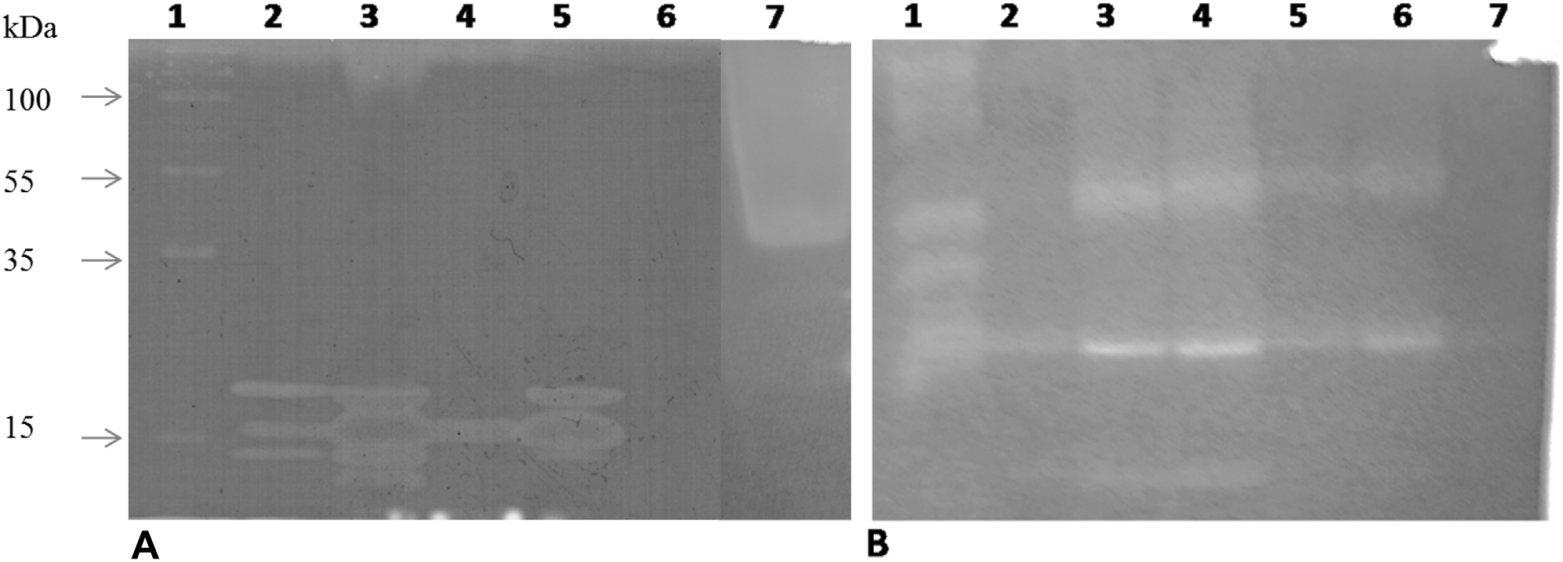
Zymograms for detection of extracellular xylanases and cellulases from solid-state cultures of H1 in different natural substrates. (A) Native gel for xylanases. Line 1: Molecular Weight marker. Line 2: Culture in wheat straw. Line 3: Culture in. maize stover Line 4: Culture in. sugarcane bagasse Line 5: Culture in. agave fibre Line 6: Culture in glucose. Line 7: Positive control. Xylanasas from *Trichoderma viridae.* (B) Native gel for cellulases. Line 1: Positive control. Cellulases from *Trichoderma viridae.* Line 2: Culture in glucose. Line 3: Culture in wheat straw. Line 4: Culture in maize stover. Line 5: Culture in sugarcane bagasse. Line 6: Culture in agave fibre. Line 7: Culture in sawdust. The arrows indicate bands with activity.

We were interested in testing this fungal strain for enzyme production of lignocellulosic substrates in similar conditions to those found in the field, so solid state fermentations were set up in the absence of NaCl. Wheat straw, maize stover and agave fibres (in that order) were the substrates where the best cellulase and xylanase activities were obtained during solid-state fermentation. Xylanase production was favoured over cellulase production when these substrates were used, and it was higher in wheat straw and maize stover (Figure 5). When a PAGE native gel with embedded xylan was stained with Congo red, four bands of approximately 10, 12, 15 and 20 kDa with xylanase activity were observed for the maize stover fermentation (Figure 6A, lane 3). The 12, 15 and 20 kDa bands also are induced, although in different proportions, when wheat straw and agave fivers were used as substrates, being the 15 kDa band very abundant in the agave fibres fermentation (Figure 6A, lanes 2 and 5). In the sugar cane bagasse fermentation only the 15 kDa band was observed (Figure 6A, lane 4). Again, no xylanase activity was observed in the zymogram when glucose was used as the carbon source Figure 6A, lane 6), indicating a repression by this sugar for xylanases expression. Distinct band patterns were observed for different substrates suggesting a differential expression of xylanases according to the substrate used.

In a native gel with cellulose stained with Congo red, we observed three bands with cellulase activity for wheat straw and maize stover cultures, while only two bands were evident in sugarcane bagasse and agave fibres (Figure 6B). Note that in cultures with glucose as a carbon source, only a low basal expression of cellulases is observed (Figure 6B), suggesting again that glucose represses the expression of most of the isoforms of these enzymes. Sawdust proved the worst substrate for induction of these enzymes (Figure 5).

*A. caesiellus* H1 also showed strong Mn peroxidase activity (Figure 5). No statistically significant differences in the Mn peroxidase activity measured in the cultures grown on wheat straw, maize stover and sawdust were observed. In the latter substrates the best Mn peroxidase activity was obtained (Figure 5). Cultures of the fungus in agave fibres and sugarcane bagasse produced lower Mn peroxidase activity with statistically significant differences between them (Figure 5).

Fungal laccases have been widely studied for their industrial applications. However, no laccase activity for H1 strain was detected in any of the conditions we tested.

Wheat straw, maize stover and agave fibres were the substrates where the higher esterase and lipase/esterase activity were determined (Figure 5). This fungus showed a very high lipase/esterase activity with 2-naphthyl acetate as a substrate in wheat straw and maize stover (Figure 5). In general, the substrates where the best enzymatic activities were observed were wheat straw and maize stover. These results support the potential of this fungus to degrade lignocellulosic material and its potential biotechnological applications. Experiments are under way to test H1 for lignocellulosic substrates degradation in the presence of NaCl.

### Optimum temperature and thermo-stability of cellulases activity

**Figure 7:**
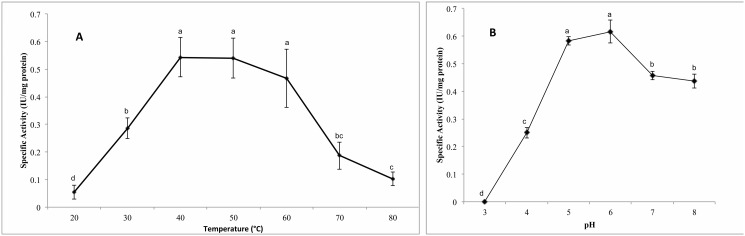
(A) Optimal temperature of celullase activity of H1. (B) Optimal pH of celullase activity of H1. Different letters indicate different statistical orders.

No statistically significant differences in the cellulase activity of the fungus at 50, 60 and 70°C were observed (Figure 7A). When fungal supernatant was incubated for one hour at 60°C, it kept 40% of the cellulases activity (residual activity). This result suggests that *A. caesiellus* cellulases are thermostable. One of the important factors for the industrial application of cellulolytic enzymes is its sturdiness [57], [122]–[125]. Our results are comparable with those obtained by Liu *et al.* [95] and Raddadi *et al.* [126], and different than those reported by Pang *et al.* [127], since they lost the cellulase activity when supernatant was heated for one hour at 50°C. In our case, the residual activity decreases to 22% (residual activity) when the supernatants were exposed for one hour at 70°C. A similar result was reported by Narra *et al.* [128], who found a dramatic decrease of the cellulolytic activity at this temperature.

### Cellulase Ph Optimum

The best cellulase activity was observed at pH values between 5 and 6 (Figure 7B). This result is consistent for other cellulases [95], [128]. However, some cellulases show optimal pH in the neutral or basic range [127], [129].

In this work we have described the isolation and characterization of a moderate halophile *A. caesiellus* strain that produces thermostable cellulases and other biotechnological interesting activities. Our results show that this strain has a great potential for lignocellulose degradation and could be used for biorefinery applications.

## Supporting Information

Figure S1 **Phylogenies for molecular taxonomic identification of moderate halophile strain H1.** (A) Molecular phylogeny considering the sequence of the fragment of the 18S ribosomal DNA. (B) Molecular phylogeny considering the sequence of the regions of the ITS1 region.
(TIF)
