# Supplementary figures and images for: Characterization of Lignocellulolytic Activities from a Moderate Halophile Strain of Aspergillus caesiellus Isolated from a Sugarcane Bagasse Fermentation
Source: PLoS One. 2014 Aug 27;9(8):e105893. doi: 10.1371/journal.pone.0105893 (PMC4146556; doi:10.1371/journal.pone.0105893)

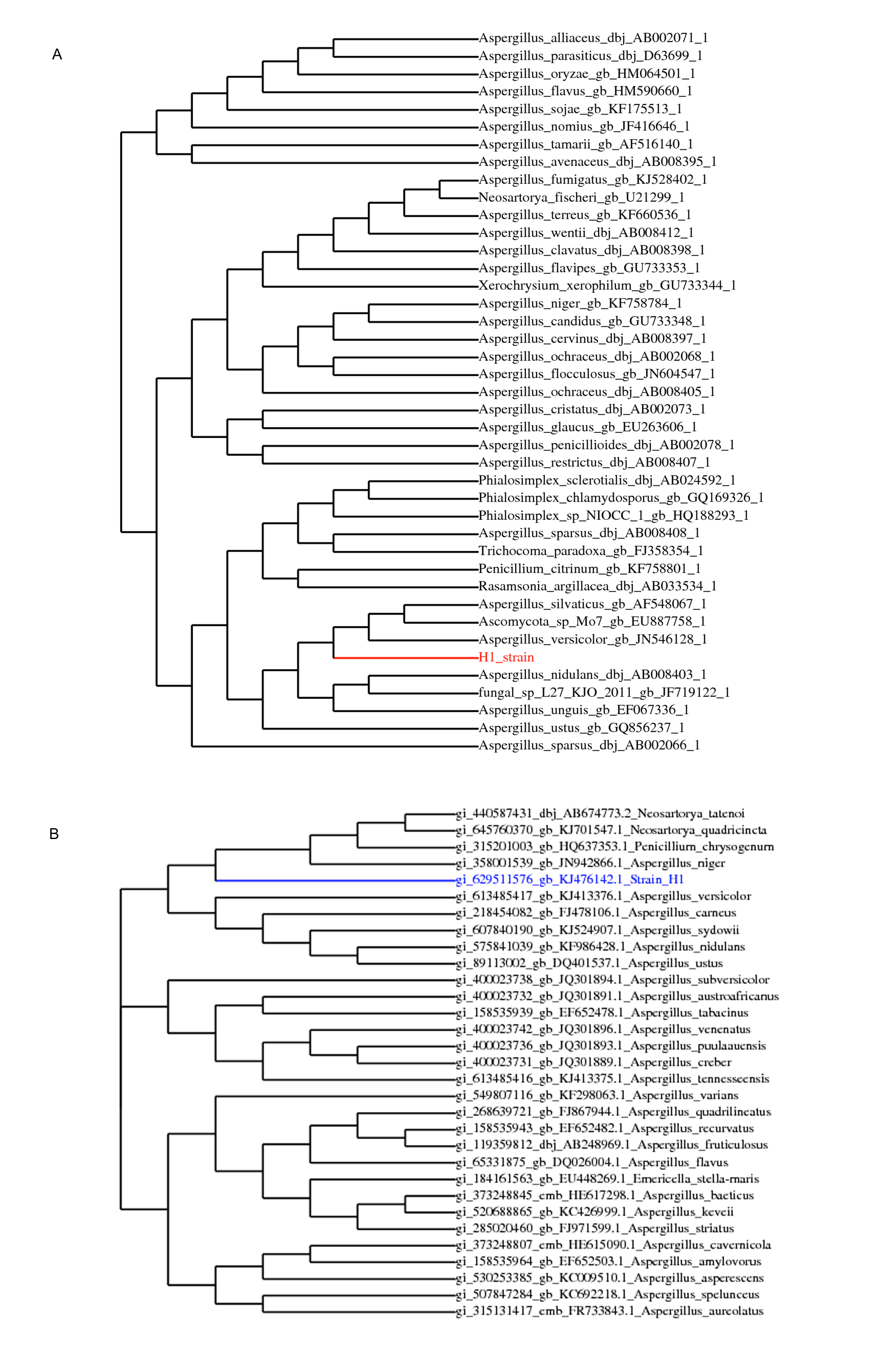

Supplement: Figure S1 — Phylogenies for molecular taxonomic identification of moderate halophile strain H1. (A) Molecular phylogeny considering the sequence of the fragment of the 18S ribosomal DNA. (B) Molecular phylogeny considering the sequence of the regions of the ITS1 region. (TIF) [file pone.0105893.s001.tif]
